# Significance of nitrosative stress and glycoxidation products in the diagnosis of COVID-19

**DOI:** 10.1038/s41598-024-59876-w

**Published:** 2024-04-22

**Authors:** Blanka Wolszczak-Biedrzycka, Justyna Dorf, Joanna Matowicka-Karna, Marzena Wojewódzka-Żeleźniakowicz, Piotr Żukowski, Anna Zalewska, Mateusz Maciejczyk

**Affiliations:** 1 Department of Psychology and Sociology of Health and Public Health, University of Warmia and Mazury in Olsztyn, 10-900 Olsztyn, Poland; 2 Department of Clinical Laboratory Diagnostics, Medical University of Bialystok, 15-089 Białystok, Poland; 3 Department of Emergency Medicine and Disasters, Medical University of Bialystok, 15-089 Białystok, Poland; 4 Department of Restorative Dentistry, Croydon University Hospital, 530 London Road, Croydon, Surrey, CR7 7YE UK; 5 Independent Laboratory of Experimental Dentistry, Medical University of Bialystok, 15-089 Białystok, Poland; 6 Department of Hygiene, Epidemiology and Ergonomics, Medical University of Bialystok, 15-089 Białystok, Poland

**Keywords:** Glycoxidation products, Nitrosative stress, COVID-19, SARS-CoV-2 virus, MEWS, Cell biology, Immunology

## Abstract

Nitrosative stress promotes protein glycoxidation, and both processes can occur during an infection with the SARS-CoV-2 virus. Therefore, the aim of this study was to assess selected nitrosative stress parameters and protein glycoxidation products in COVID-19 patients and convalescents relative to healthy subjects, including in reference to the severity of COVID-19 symptoms. The diagnostic utility of nitrosative stress and protein glycoxidation biomarkers was also evaluated in COVID-19 patients. The study involved 218 patients with COVID-19, 69 convalescents, and 48 healthy subjects. Nitrosative stress parameters (NO, S-nitrosothiols, nitrotyrosine) and protein glycoxidation products (tryptophan, kynurenine, N-formylkynurenine, dityrosine, AGEs) were measured in the blood plasma or serum with the use of colorimetric/fluorometric methods. The levels of NO (*p* = 0.0480), S-nitrosothiols (*p* = 0.0004), nitrotyrosine (*p* = 0.0175), kynurenine (*p* < 0.0001), N-formylkynurenine (*p* < 0.0001), dityrosine (*p* < 0.0001), and AGEs (*p* < 0.0001) were significantly higher, whereas tryptophan fluorescence was significantly (*p* < 0.0001) lower in COVID-19 patients than in the control group. Significant differences in the analyzed parameters were observed in different stages of COVID-19. In turn, the concentrations of kynurenine (*p* < 0.0001), N-formylkynurenine (*p* < 0.0001), dityrosine (*p* < 0.0001), and AGEs (*p* < 0.0001) were significantly higher, whereas tryptophan levels were significantly (*p* < 0.0001) lower in convalescents than in healthy controls. The ROC analysis revealed that protein glycoxidation products can be useful for diagnosing infections with the SARS-CoV-2 virus because they differentiate COVID-19 patients (KN: sensitivity—91.20%, specificity—92.00%; NFK: sensitivity—92.37%, specificity—92.00%; AGEs: sensitivity—99,02%, specificity—100%) and convalescents (KN: sensitivity—82.22%, specificity—84.00%; NFK: sensitivity—82,86%, specificity—86,00%; DT: sensitivity—100%, specificity—100%; AGE: sensitivity—100%, specificity—100%) from healthy subjects with high sensitivity and specificity. Nitrosative stress and protein glycoxidation are intensified both during and after an infection with the SARS-CoV-2 virus. The levels of redox biomarkers fluctuate in different stages of the disease. Circulating biomarkers of nitrosative stress/protein glycoxidation have potential diagnostic utility in both COVID-19 patients and convalescents.

## Introduction

COVID-19, an infection caused by the severe-acute-respiratory-syndrome-related coronavirus 2 (SARS-CoV-2), can be asymptomatic or can produce mild or severe symptoms that can lead to life-threatening multiorgan failure in patients^1,2^. The severity of symptoms is evaluated with the use of diagnostic scales, including the Modified Early Warning Score (MEWS)^3,4^ An infection with the SARS-CoV-2 virus triggers excessive production of reactive oxygen species (ROS)^5^ and reactive nitrogen species (RNS)^6^, which enhances protein glycoxidation^7,8^. In patients who had experienced severe symptoms, as well as mild and moderate symptoms of COVID-19, these processes are intensified and can lead to various complications^7^. Cardiovascular^9^, muscular^10^, and neurological problems^11^ are the most common complications. COVID-19 not only increases the risk of new diseases, but it can also aggravate pre-existing comorbidities^12^.

Under physiological conditions, ROS and RNS participate in cell signaling and play an important role in defense against pathogens^13^. Nuclear factor kappa-light-chain-enhancer of activated B cells (NF-κB)^14^ and nuclear erythroid-2 related factor 2 (Nrf2)^15^, which are responsible for maintaining the redox balance in cells and generating a response to an inflammation^16^. The SARS-CoV-2 virus enters host cells via the angiotensin-converting enzyme 2 (ACE2) receptor^17^, which decreases the expression of the angiotensin II type-2 receptor and the placental growth factor (PlGF), and increases the expression of the angiotensin II type-1 receptor and soluble fms-like tyrosine kinase-1 (sFlt-1)^18,19^. These processes can disrupt mitochondrial function and trigger the overproduction of ROS, including O_2_^−^, and RNS. When the antioxidant defense system is weakened, the above can lead to thrombophilia and hypoxia^20,21^.

In COVID-19 patients, non-enzymatic glycation of the ACE2 receptor is intensified, which increases the receptor's susceptibility to interactions with the SARS-CoV-2 spike (S) protein^17^. Non-enzymatic glycation of ACE2 modifies amino acids and induces changes in the protein’s tertiary structure, and this mechanism is implicated in the pathogenesis of COVID-19^22^. In turn, an increase in the levels of advanced glycation end products (AGEs) is associated with severe COVID-19 and increased incidence of complications^22,23^.

Tryptophan, the precursor of nicotinamide adenine dinucleotide (NAD^+^), plays an important role in the redox balance, and this amino acid should be considered in analyses of the redox imbalance during COVID-19^24^. Tryptophan is absorbed via the ACE2 transport pathway^25^. This amino acid is metabolized along serotonin and kynurenine pathways, and in severe COVID-19, these pathways are disrupted, which exacerbates oxidative/nitrosative stress^26^. Hyperactivation of the kynurenine pathway caused by the SARS-CoV-2 weakens the host’s immune response^27^. In addition, the accumulation of tryptophan and its metabolites, including N-formylkynurenine (NFK), kynurenine (KN), and dityrosine (DT), in COVID-19 patients promotes inflammation and contributes to organ failure^28^.

A better understanding of the pathogenesis, progression, and complications associated with the SARS-CoV-2 infection can contribute to the development of new diagnostic methods and effective therapeutic protocols for COVID-19 patients. Researches showed that biomarkers are really useful in different diseases^29–31^. We expect to observe changes in the concentration of nitrosative stress biomarkers and protein glycoxidation products infected with the SARS-CoV-2 virus. Therefore, the aim of this study was to evaluate those biomarkers in patients infected with the SARS-CoV-2 virus and COVID-19 convalescents. Changes in the examined parameters were also analyzed in patients with different severity of COVID-19. The diagnostic utility of selected biomarkers was assessed in COVID-19 patients and convalescents in the ROC analysis.

## Results

### Characteristics of COVID-19 patients

The study group involved 218 COVID-19 patients (115 men and 103 women). The length of hospital stay was less than 10 days in 194 patients, 10 to 20 days in 13 patients, and more than 20 days in 6 patients. Comorbidities were diagnosed in 107 subjects, including hypertension (48 patients), heart failure (29 patients), and diabetes (24 patients). The study group was divided into four subgroups with different COVID-19 severity based on the MEWS (Table 1): MEWS 1–106 patients, MEWS 2–70 patients, MEWS3–28 patients, and MEWS 4–14 patients. The study group is described in detail in Tables 2, 3.

**Table 1:** Modified Early Warning Score (MEWS).

Score	MEWS 1	MEWS 2	MEWS 3	MEWS 4
Respiratory rate, breaths/min	9–14	15–20	21–29 or ≤ 8	> 29
Heart rate, bpm	51–100	101–110 or 41–50	111–129 or ≤ 40	> 129
Systolic blood pressure, mm Hg	101–199	81–100	≤ 200 or 71–80	≤ 70
Hourly urine, mL/kg of body weight/h	> 0.5		< 0.5	Nil
Body temperature, °C	36.1–38	38.1–38.5 or 35.1–36	≤ 38.6 or ≤ 35	
Neurological symptoms	Alert	Responsive to voice	Responsive to pain	Unresponsive

**Table 2:** Characteristics of COVID-19 patients.

	All patients with COVID-19	COVID-19 severity according to MEWS			
Clinical features	n (%)	1	2	3	4
Number of patients	218	106 (49.8%)	70 (32.9%)	28 (13.1%)	14 (4.2%)
Age					
≤ 55	93 (44.13%)	37 (34.91%)	37 (52.86%)	15 (53.57%)	7 (55.56%)
56–75	67 (30.52%)	33 (31.13%)	20 (28.57%)	9 (32.14%)	5 (33.33%)
> 76	54 (25.35%)	36 (33.96%)	13 (18.57%)	4 (14.29%)	2 (11.11%)
Sex					
Female	103 (46.95%)	51 (48.11%)	33 (47.14%)	12 (42.86%)	6 (44.44%)
Male	115 (53.05%)	55 (51.89%)	37 (52.86%)	16 (57.14%)	8 (55.56%)
Hospitalization time					
≤ 10 days	197 (91.08%)	96 (90.57%)	63 (90%)	27 (96.43%)	12 (88.89%)
10–20 days	15 (6.10%)	8 (7.55%)	4 (5.71%)	1 (3.57%)	0
> 20 days	6 (2.82%)	2 (1.88%)	3 (4.29%)	0	2 (11.11%)
Comorbidities (n,%)					
Absent	108 (49.77%)	47 (44.34%)	38 (54.29%)	16 (57.14%)	8 (55.56%)
Present	110 (50.23%)	59 (55.66%)	32 (45.71%)	12 (42.86%)	3 (44.44%)
Hypertension	46 (22.54%)	27 (25.47%)	18 (25.71%)	2 (7.14%)	1 (11.11%)
Diabetes mellitus	22 (11.27%)	16 (7.51%)	6 (8.57%)	2 (7.14%)	0
Obesity	5 (3.29%)	2 (1.89%)	4 (5.71%)	1(3.57%)	0
Heart failure	26 (15.62%)	21 (19.81%)	5 (7.14%)	0	2 (33.33%)
Other (e.g. cancer, hematological disorders)	11 (6.10%)	4 (1.88%)	6 (8.57%)	3 (10.71%)	0
Cough					
Yes	59 (26.76%)	19 (17.92%)	26 (37.14%)	10 (35.71%)	4 (22.22%)
No	159 (73.24%)	87 (82.08%)	44 (62.86%)	18 (64.29%)	10 (77.78%)
Fever					
Yes	99 (45.54%)	103 (97.1%)	65 (92.86%)	21 (75%)	12 (88.89%)
No	119 (54.46%)	3 (2.9%)	5 (7.14%)	7 (25%)	2 (11.11%)
Dyspnea					
Yes	63 (28.64%)	23 (21.70%)	21 (30%)	10 (35.71%)	11 (77.78%)
No	155 (71.36%)	83 (78.30%)	49 (70%)	18 (64.29%)	3(22.22%)
Gastrointestinal symptoms					
Yes	7 (2.35%)	3 (2.83)%	1 (1.43%)	1 (3.57%)	0
No	211 (97.65%)	103 (97.17%)	69 (98.57%)	27 (96.43%)	0
Respiratory failure					
Yes	10 (3.76%)	3 (2.83%)	3 (4.28%)	1(3.57%)	2 (11.11%)
No	208 (96.24%)	103 (97.17%)	67 (95.72%)	27 (96.43%)	12 (88.89%)

*ALT* alanine aminotransferase, *AST* aspartate aminotransferase, *CRP* C-reactive protein, *INR* international normalized ratio, *PLT* platelet count, *RBC* red blood cell count, *WBC* white blood cell count.

**Table 3:** A comparison of selected blood test results in COVID-19 patients with different MEWS scores.

COVID-19 severity	MEWS 1	MEWS 2	MEWS 3	MEWS 4
WBC	7.90 (2.73–39.24)	6.53 (2.78–14.62)	7.48 (1.41–16.11)	10.41 (3.91–22.52)
RBC	4.50 (2.32–6.28)	4.53 (2.4–6.28)	4.46 (1.14–5.37)	4.61 (4.16–5.55)
PLT	221 (55–595)	209 (56–620)	257 (71–490)	286 (98–525)
CRP	57.36 (0–289.9)	69.91 (1–303)	93 (1–354.8)	150.57 (1–228.5)
AST	45 (0–141)	53 (15–176)	61 (13–204)	54 (21–84)
ALT	40 (0–162)	48 (9–206)	45 (10–161)	46.2 (14–93)
Creatinine	1.2 (0.41–10.39)	1.08 (0.54–10.2)	1.27 (0.50–12.12)	0.98 (0.5–1.81)
Glucose	130 (69–396)	120 (128–144)	111 (77–209)	127 (88–180)
Na	137 (102–145)	137 (128–144)	138 (128–145)	137 (127–141)
K	4.36 (2.9–7.1)	4.31 (3–6.7)	4.25 (3.3–5.3)	4.08 (3.3–5.1)
INR	1.15 (0.89–2.67)	1.14 (0.88–2.82)	1.19 (0.9–3.55)	1.17 (1.02–1.39)
D-dimers	3.27 (0–20)	1.50 (0.27–7.01)	2.94 (0.27–20)	2.33 (0.37–4.84)

*ALT* alanine aminotransferase, *AST* aspartate aminotransferase, *CRP* C-reactive protein, *INR* international normalized ratio, *PLT* platelet count, *RBC* red blood cell count, *WBC* white blood cell count.

#### A comparison of nitrosative stress parameters in patients infected with the SAR-CoV-2 virus, convalescents, and healthy controls, including in reference to the severity of COVID-19

**Figure 1:**
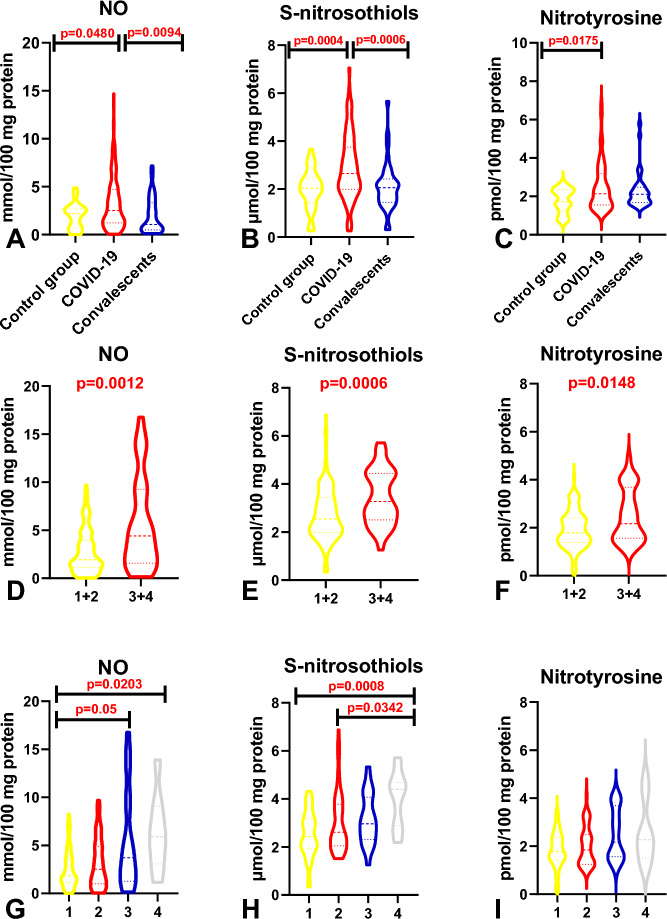
Comparison of nitrosative stress biomarkers: in COVID-19 patients, convalescents, and the control group (**A**, **B**, **C**); in COVID-19 patients with mild/moderate (1 + 2) and moderately severe/severe (3 + 4) symptoms (**D**, **E**, **F**); in COVID-19 patients with different severity of symptoms assessed based on the MEWS (**G**, **H**, **I**). Key: *NO* nitric oxide.

The analysis revealed that NO, S-nitrosothiol, and nitrotyrosine (NT) levels were significantly higher in COVID-19 patients than in the control group (*p* = 0.0480, *p* = 0.0004, *p* = 0.0175). In addition, NO and S-nitrosothiol concentrations increased significantly in COVID-19 patients relative to the group of convalescents (*p* = 0.0094, *p* = 0.0006) (Fig. 1A–C). A comparison of patients with mild/moderate and moderately severe/severe symptoms demonstrated that all nitrosative stress parameters were significantly higher in MEWS 3 + 4 subgroups than in MEWS 1 + 2 subgroups (NO *p* = 0.0012, S-nitrosothiols *p* = 0.006, nitrotyrosine *p* = 0.0148) (Fig. 1D–F). Nitric oxide levels were significantly higher in MEWS 3 and MEWS 4 patients than in MEWS 1 patients (*p* = 0.05), and significantly higher in MEWS 4 patients than in MEWS 1 patients (*p* = 0.0203). The concentration of S-nitrosothiols was also significantly higher in the MEWS 4 subgroup than in MEWS 1 (*p* = 0.0008) and MEWS 2 (*p* = 0.0342) patients (Table 4, Fig. 1G–I).

**Table 4:** Comparison of the levels nitrosative stress parameters in COVID-19 patients with different severity of disease symptoms (MEWS 1 + MEWS 2 vs MEWS 3 + MEWS 4).

Parameter	MEWS 1	MEWS 2	MEWS 3	MEWS 4	*p*-value
	Median (min–max)	Median (min–max)	Median (min–max)	Median (min–max)	
Nitrosative stress					
NO	1.841 (0.02524–8.229)	2.516 (0.04551–9.680)	3.713 (0.1349–16.75)	5.897 (1.149–13.89)	0.0042
S-nitrosothiols	2.430 (0.3386–4.319)	2.612 (1.512–6.873)	2.963 (1.257–5.328)	4.406 (2.187–5.712)	0.0005
Nitrotyrosine	1.774 (0.1762–3.531)	1.839 (0.9340–4.110)	2.166 (1.160–4.096)	2.270 (1.110–4.832)	0.1077
Protein glycoxidation products					
Tryptophan	1656 (847.4–2378)	1625 (818.4–2249)	1564 (675.4–2012)	1459 (1015–1713)	0.0428
KN	1423 (120.1–2730)	1476 (337.8–2914)	1769 (1067–3745)	2071 (1085–3812)	0.0279
NFK	817.0 (55.02–1739)	838.1 (49.65–2081)	1025 (48.54–2540)	1238 (362.1–3088)	0.0935
DT	663.5 (158.4–1560)	701.0 (229.5–2312)	779.0 (371.1–2280)	975.5 (508.5–3239)	0.0403
AGEs	72.88(26.22–146.5)	95,58(28,77–250)	93,7(29,44–554,1)	86,75(12,5–577,4)	0.2987

*AGEs* advanced glycation end products, *DT* dityrosine, *KN* kynurenine, *NFK* N-formylkynurenine, *NO* nitric oxide.

#### A comparison of the levels protein glycoxidation products in patients infected with the SAR-CoV-2 virus, convalescents, and healthy controls, including in reference to the severity of COVID-19

**Figure 2:**
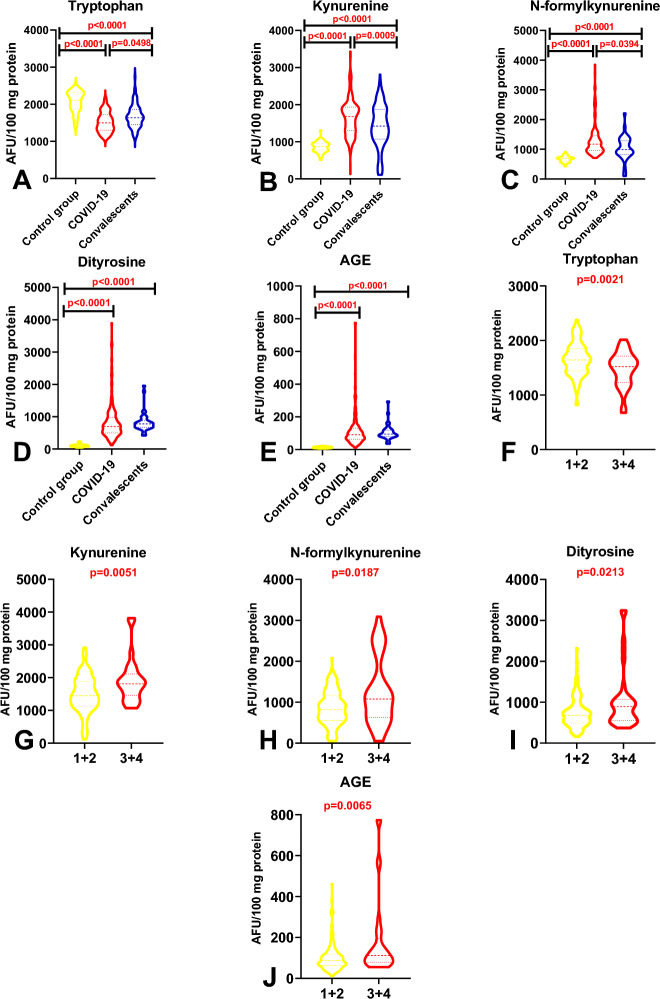
Comparison of the levels protein glycoxidation products: in COVID-19 patients, convalescents, and the control group (**A**, **B**, **C**, **D**, **E**); in COVID-19 patients with mild/moderate (1 + 2) and moderately severe/severe (3 + 4) symptoms (**F**, **G**, **H**, **I**, **J**). Key: *AGEs* advanced glycation end products.

Tryptophan concentration was significantly lower in COVID-19 patients than in the control group (*p* < 0.0001) and convalescents (*p* = 0.0498), and significantly lower in convalescents than in the control group (*p* < 0.0001). In turn, KN, NFK, DT, and AGE levels were significantly higher in COVID-19 patients than in healthy controls (*p* < 0.0001), and significantly higher in convalescents than in the control group (*p* < 0.0001). In addition, KN and NFK levels were higher in patients infected with the SARS-CoV-2 virus than in the control group (*p* = 0.0009, *p* = 0.0394) (Fig. 2A–E). An analysis of protein glycoxidation products revealed no significant differences in COVID-19 patients with different severity of symptoms evaluated based on the MEWS (Table 4). However, tryptophan concentration decreased, whereas KN, NFK, and DT concentrations increased with the aggravation of disease symptoms (Table 4). In turn, a comparison of patients with mild/moderate symptoms and moderately severe/severe symptoms revealed that the concentrations of all protein glycoxidation products were higher in MEWS 3 + 4 subgroups than in MEWS 1 + 2 subgroups (tryptophan *p* = 0.0021, KN *p* = 0.0051, NFK = 0.0187, dityrosine *p* = 0.0213, AGEs *p* = 0.0065) (Fig. 2E,G–J).

### ROC analysis

The diagnostic utility of nitrosative stress parameters and protein glycoxidation products as biomarkers during an infection with the SARS-CoV-2 virus was assessed in the ROC analysis (Tables 5 and 6). The analysis revealed that protein glycoxidation products can be useful for differentiating between patients infected with the SARS-CoV-2 virus and healthy subjects (AUC for tryptophan = 0.9083, for KN = 0.9594, for NFK = 0.9868, for AGE = 0.9971). In addition, tryptophan (AUC = 0.8241), KN (AUC = 0.8546), NFK (AUC = 0.8697), DT (AUC = 1.000), and AGEs (AUC = 1.000) were also useful diagnostic biomarkers for differentiating between convalescents and the control group (AUC = 1.000) (Table 5).

**Table 5:** Area under the curve (AUC) values of nitrosative stress biomarkers and protein glycoxidation products that were used to differentiate between COVID-19 patients and the control group (A); COVID-19 patients and convalescents (B); convalescents and the control group (C).

Parameter	AUC	*p*-value	Cut-off	Sensitivity (%)	Specificity (%)	95% confidence interval
A. COVID-19 vs control						
S-nitrosothiols	0.7062	< 0.0001	2.288	64.39	64.37	0.6257–0.7867
Tryptophan	0.9083	< 0.0001	1822	80	80	0.8427–0.9740
KN	0.9594	< 0.0001	1050	91.20	92.00	0.9357–0.9827
NFK	0.9868	< 0.0001	818.9	92.37	92.00	0.9745–0.9991
AGE	0.9971	< 0.0001	22,70	99,02	100	0.9917–1.000
B. COVID-19 vs convalescents						
NO	0.6563	0.0032	1.903	57.14	57.50	0.5575–0.7751
S-nitrosothiols	0.6930	0.0002	2.241	63.89	63.90	0.6061–0.7798
Tryptophan	0.6473	0.0100	1584	60.34	60.00	0.5389–0.7558
NFK	0.6450	0.0093	1075	60.00	58.47	0.5371–0.7530
C. Convalescents vs control						
Tryptophan	0.8241	< 0.0001	1866.00	77.01	77.5	0.7523–0.8959
KN	0.8546	< 0.0001	974.9	82.22	84.00	0.7957–0.8955
NFK	0.8697	< 0.0001	744.7	82.86	86.00	0.7707–0.9688
DT	1.000	< 0.0001	327.1	100	100	1.000–1.000
AGEs	1.000	< 0.0001	29,10	100	100	1,000–1,000

*AGEs* advanced glycation end products, *DT* dityrosine, *KN* kynurenine, *NFK* N-formylkynurenine, *NO* nitric oxide.

**Table 6:** Area under the curve (AUC) values of nitrosative stress biomarkers and protein glycoxidation products that were used to differentiate between COVID-19 patients with different severity of symptoms (MEWS 1 + MEWS2 vs MEWS 3 + MEWS 4).

Parameter	AUC	*p*-value	Cut-off	Sensitivity (%)	Specificity (%)	95% confidence interval
NO	0.6798	0.0014	2.786	59.38	60.40	0.5666–0.7931
S-nitrosothiols	0.6832	0.0007	2.871	61.11	61.48	0.5860–0.7805
Tryptophan	0.6512	0.0085	1598	58.06	58.87	0.5457–0.7567
KN	0.6714	0.0055	1680	65.38	64.38	0.5670–0.7759

*KN* kynurenine, *NO* nitric oxide.

## Discussion

According to the literature, oxidative and nitrosative stress influences the pathogenesis and severity of infections with the SARS-CoV-2 virus^1,32,33^, and the present study was undertaken to evaluate nitrosative stress parameters (NO, S-nitrosothiols, and NT) and protein glycoxidation products (tryptophan, KN, NFK, DT, and AGEs) in COVID-19 patients and convalescents, and to examine the above parameters in COVID-19 patients with different severity of disease symptoms evaluated based on the MEWS. The concentrations of NO, NT, S-nitrosothiols, KN, NFK, DT, and AGEs were higher, whereas tryptophan concentration was lower in COVID-19 patients than in the control group and convalescents. The study demonstrated that in patients with a history of COVID-19, the levels of KN, NFK, DT, and AGEs were significantly higher, whereas tryptophan concentration was significantly lower than in persons who had never been infected with the SARS-CoV-2 virus. Significant differences in nitrosative stress parameters and protein glycoxidation products were observed among COVID-19 patients with different disease severity. In addition, protein glycoxidation products were useful diagnostic biomarkers for differentiating COVID-19 patients and convalescents from healthy subjects. This is the first study to analyze changes in the levels of nitrosative stress and protein glycoxidation products in convalescents, and it provides valuable information for predicting long-term consequences of COVID-19.

During COVID-19, ROS and RNS are overproduced, which contributes to oxidative and nitrosative stress^32^. In the human body, nitric oxide (NO), one of the main RNS, is synthesized from L-arginine in a reaction catalyzed by the nitric oxide synthase (NOS) family of enzymes, including neuronal NOS (nNOS), endothelial NOS (eNOS), and inducible NOS (iNOS)^34,35^. These enzymes have been identified in inflammatory cell infiltrates^36^. Under physiological conditions, NO plays a number of positive roles by relaxing vascular and bronchial smooth muscles, inhibiting platelet aggregation, participating in neurotransmission and immune processes^37–39^. In turn, excess NO exerts proinflammatory effects and can contribute to bronchial hyperreactivity and pulmonary endothelial damage, which has been observed in COVID-19 patients^40,41^. In the host organism, the infectious process is initiated when the S protein of the SARS-CoV-2 virus binds to transmembrane protease serine 2 (TMPRSS2) and ACE2^42^. In the next stage, the virus infects endothelial cells, which causes angiotensin II to bind to the angiotensin II receptor type 1 (AT-1R), activates nicotinamide adenine dinucleotide phosphate (NADPH) oxidase, and increases ROS production^43^. The above leads to the activation of various signaling pathways, which increases the production of proinflammatory interleukins. Cytokines decrease the availability of endogenous NO, prostaglandin and its analogs, which leads to endothelial dysfunction and damage^44^. In addition, increased production of proinflammatory markers and prothrombotic factors increases the risk of serious chronic complications not only in the respiratory tract, but also in vascular and nervous systems^37,45^. In the present study, NO levels were higher in COVID-19 patients than in the control group and convalescents, and they tended to increase with the severity of disease symptoms. Research has shown that an imbalance of NO and RNS is associated with lung damage. Nitric oxide is a proinflammatory mediator of lung damage in COVID-19 patients^46^. In this study, NO levels were lowest in convalescents, which corroborates the hypothesis that the SARS-CoV-2 virus impairs mitochondrial function, intensifies oxidative and nitrosative stress, and may initiate a feedback loop that contributes to chronic inflammation and endothelial damage long after viral particles have been eliminated from the body.

Increased production of NO and other RNS leads to posttranslational modification of proteins, including S-nitrosylation, glutathionylation, and tyrosine nitration^47^. S-nitrosylation is a process of selective covalent post-translation modification which adds a nitrosyl group to a thiol/sulfhydryl group of cysteine and leads to the formation of the S-nitrosothiol (RSNO) derivative^48^. S-nitrosylation exerts protective effects by preventing ROS from inducing further irreversible changes in critical protein thiols^49^. According to research, S-nitrosylation can play an important role in COVID-19 by acting as a defense mechanism^50^. In the current study, S-nitrosothiol levels were higher in COVID-19 patients than in the control group and convalescents, which indicates that the S-nitrosylation mechanism had been activated to protect the body against the virus. Other authors observed that S-nitroso-N-acetylpenicillamine (SNAP), an NO donor, increases the survival of host cells after the infection and inhibits the replication of the severe-acute-respiratory-syndrome coronavirus 1 (SARS-CoV-1) at the RNA and cellular level^51^. Research has also shown that NO prevents the SARS-CoV-2 virus from entering cells and replicating^41^. In the present study, the increase in S-nitrosothiol levels in COVID-19 patients with moderately severe/severe symptoms, and persistently elevated S-nitrosothiol concentrations in convalescents could be associated with more serious complications in these groups.

Reactive nitrogen species can also induce a two-step tyrosine nitration reaction^52^. In the present study, nitrotyrosine (NT) levels were higher in COVID-19 patients than in healthy controls and tended to increase with the severity of disease symptoms. The presence of NT in proteins is associated with the rapid modification of proteins under nitrosative stress, which contributes to pro-oxidative processes^53^. In addition, tyrosine nitration is mediated by peroxynitrite (ONOO^−^) which is produced in a reaction between NO and O_2_^−^. It should be noted that ONOO is much more reactive and toxic than NO, and it is a potent oxidant that reacts directly with sulfhydryl, iron-sulfur, and zinc-thiolate complexes, and participates in oxidation and hydroxylation reactions^54^. Protein nitration occurs under physiological conditions, and it regulates many biological processes, including energy metabolism, signal transduction, apoptosis and cell death, enzyme inactivation, protein degradation, mitochondrial dysfunctions, and immunogenicity^55^. In the present study, nitrotyrosine levels were higher in COVID-19 patients than in the control group, and higher in patients with moderately severe/severe symptoms than in patients with mild/moderate symptoms, which indicates that nitrosative stress was enhanced in these groups. Similar observations were made by other authors^56^.

The levels of protein glycoxidation products (KN, NFK, DT, and AGEs) were significantly higher, whereas tryptophan concentration was significantly lower in patients infected with the SARS-CoV-2 virus and in convalescents than in the control group (*p* < 0.0001). In addition, tryptophan concentration was significantly lower, whereas the levels of KN and NFK were higher in COVID-19 patients than in convalescents. A significant decrease in tryptophan fluorescence and an increase in the remaining glycoxidation products was also noted in COVID-19 patients with moderately severe/severe symptoms (MEWS3 + MEWS4) relative to patients with mild/moderate symptoms (MEWS1 + MEWS2). The results of the ROC analysis indicate that tryptophan, KN, NFK, and AGEs are highly useful diagnostic parameters for differentiating between COVID-19 patients and healthy subjects (AUC = 0.9083, 0.9594, 0.9868, and 0.9971, respectively), and together with DT, these parameters are useful for differentiating between convalescents and healthy controls (AUC = 0.8241, 0.8546, 0.8697, 1.000). In the authors' previous research, the levels of proinflammatory cytokines were elevated during COVID-19^3,57,58^. Increased production of IFN-γ, IL-1β, IL-6, and ROS induces indoleamine 2,3-dioxygenase (IDO) which mediates tryptophan catabolism, decreases tryptophan concentration, and increases the levels of tryptophan catabolites (TRYCATs), including NFK and KN^59^.

In COVID-19 patients, other authors observed increased activity of the TRYCAT pathway^60,61^, as manifested by decreased tryptophan concentration and increased levels of KN and NFK, resulting from increased IDO activity under the influence of pro-inflammatory cytokines. In addition, the SARS-CoV-2 virus stimulates the aryl hydrocarbon receptor (AhR) and increases KN levels, which leads to the systemic AhR activation syndrome (SAAR) that intensifies inflammation, induces thrombophilia, and contributes to organ damage^62,63^. Therefore, it was hypothesized that the activation of the TRYCAT pathway can aggravate COVID-19 symptoms and prolong recovery^26^. A decrease in tryptophan concentration, accompanied by an increase in the levels of its metabolites can be associated with poor prognosis not only in COVID-19 patients, but also in convalescents. It should be noted that KN levels also increase in neuropsychiatric disorders, including depression, anxiety, and psychosis^64^. Tryptophan metabolites exert pro-oxidative and neurotoxic effects, and they are associated with musculoskeletal injuries^64^. The activation of the TRYCAT pathway can aggravate pre-existing comorbidities in COVID-19 patients and convalescents^65^. The above applies particularly to patients with obesity, dementia, hypertension, heart disease, stroke, chronic obstructive pulmonary disease, and chronic kidney disease. An increase in the KN/tryptophan ratio indicates that IDO is activated in all of the above conditions^59,63^.

In the current study, the fluorescence intensity of AGEs was higher in COVID-19 patients and convalescents than in the control group, which can be associated with a poor prognosis and potential complications. An increase in the levels of AGEs contributes to inflammation, and it can also lead to diabetes and cardiovascular diseases^66,67^. According to the literature, higher levels of AGEs are associated with more severe symptoms and an increased risk of mortality in COVID-19. Advanced glycation end products act through the AGE receptor (RAGE) which is expressed mainly on the surface of type I and II pneumocytes and alveolar macrophages that are associated with acute lung injury during infections with the SARS-CoV-2 virus^68^. The authors’ previous studies^57,58^ demonstrated that when AGEs bind to the RAGE, signaling pathways, including the NF-κB pathway, are activated, which increases chemokine and cytokine levels. The S protein of the SARS-CoV-2 virus can bind to CD147, a multi-ligand glycoprotein which is synthesized in hyperglycemia and during RAGE activation^69^. CD147 is strongly expressed in type II pneumocytes, immune cells, endothelial cells, and platelets, and it also plays an important role in COVID-19-related pneumonia. The activity of matrix metalloproteinase (MMP) can increase, and cellular junctions can be destabilized when AGEs induce CD147 glycosylation in endothelial cells^70,71^. The present study also demonstrated that AGEs can be highly useful diagnostic biomarkers for differentiating between COVID-19 patients (AUC = 0.9971) and convalescents (AUC = 1.000).

In summary, the study revealed a potential relationship between nitrosative stress and protein glycoxidation during infections with the SARS-CoV-2 virus. In COVID-19 patients, the majority of changes in nitrosative stress parameters and protein glycoxidation products were consistent with those described in the literature. The levels of KN, NFK, DT, and AGEs were higher, whereas concentration was lower in convalescents (without comorbidities) than in healthy subjects. These observations suggest that COVID-19 disrupts physiological processes and induces adverse changes in redox biomarkers, which increases the risk of various post-infection complications. Protein glycoxidation products differentiate COVID-19 patients and convalescents from healthy subjects with high sensitivity and specificity. In the future, the present findings can be used in molecular analyses to evaluate the diagnostic utility of redox biomarkers in COVID-19 patients divided into similarly sized subgroups based on the severity of disease symptoms (MEWS), as well as in a larger population of convalescents. Further research is also needed to assess the relationship between nitrosative stress and COVID-19 complications. The study has several limitations. COVID-19 patients were divided into variously sized subgroups based on the MEWS. In addition, the evaluated redox parameters are also influenced by other diseases; therefore, they should be analyzed only in patients who are free of disorders associated with nitrosative stress and intensified glycation.

## Materials and methods

### Description of the study

A single-center study was conducted between January and November 2021. The study involved patients with a confirmed SARS-CoV-2 infection who were admitted to the Emergency Ward of the Clinical Hospital of the Medical University of Białystok. All participants gave their written consent to participate in the research. The study was approved by the Bioethics Committee of the Medical University of Białystok (decision No. APK.002.26.2021).

### COVID-19 patients

The study group was composed of 218 unvaccinated patients (115 men and 103 women aged 26–87 years) who tested positive for the presence of SARS-CoV-2 genetic material in nasal and throat swabs in the PCR assay (GeneXpert Cepheid). The PCR test was administered upon hospital admission, and venous blood for analysis was collected once, immediately after the patients had tested positive for COVID-19.

The severity of COVID-19 symptoms was evaluated with the use of the Modified Early Warning Score (MEWS)^72^ based on the following diagnostic parameters: respiratory rate, blood pressure, heart rate, body temperature, and neurological symptoms. The patients were divided into four groups: MEWS 1—asymptomatic and mildly symptomatic infection, MEWS 2—symptomatic infection with pneumonia without symptoms of respiratory failure, MEWS 3—symptomatic infection with pneumonia and symptoms of respiratory failure, and MEWS 4—symptomatic infection with multiple organ failure (Table 1)0.29 In addition, the study group was divided into two subgroups: patients with mild and moderate symptoms (MEWS 1 + 2) and patients with moderately severe and severe symptoms (MEWS 3 + 4).

Study group patients were also subjected to the following laboratory tests: hematological analyses (peripheral blood cell morphology), biochemical analyses (CRP, AST, ALT, creatinine, glucose, Na, K), coagulation tests (INR, D-dimers), and imaging tests (radiograph and computed tomography scan of the chest). Patient demographics, time of hospital stay (in days), comorbidities (hypertension, cancer, hematological disorders, diabetes, obesity, coronary heart disease), and clinical symptoms (gastrointestinal symptoms, fever, cough, dyspnea, acute respiratory distress syndrome) were also assessed.

### Persons with a history of COVID-19 (convalescents)

The group of convalescents consisted of unvaccinated individuals without comorbidities who tested positive for the presence of anti-SARS-CoV-2 antibodies in the blood serum. This group comprised 69 persons (30 women and 39 men aged 25–68 years). The convalescents had to meet the following inclusion criteria: a positive result of the PCR test for SARS-CoV-2 genetic material, compulsory quarantine that ended with a negative result of the PCR test at least 14 days before the study, and the absence of clinical symptoms of COVID-19. Blood for the determination of redox parameters was collected 14–30 days after a negative result of the PCR test.

### Control group

The control group consisted of 48 unvaccinated persons of both sexes (20 women and 28 men aged 26–68 years), without symptoms of infection, comorbidities, or history of COVID-19 (based on a negative result of a laboratory test confirming the absence of anti-SARS-CoV-2 antibodies in the blood serum). Control group subjects were recruited among clients who visited the LAB110 laboratory in Białystok for routine tests.

### Blood collection

Venous blood for the analysis of nitrosative and carbonyl stress parameters was collected from COVID-19 patients, convalescents, and healthy controls into S-Monovette K3 EDTA and S-Monovette®® tubes (Sarstedt, Germany). Blood was collected in a fasting state, and the participants had not performed strenuous physical activity for 24 h before the test. The collected samples were immediately centrifuged at 4000×*g* for 10 min at a temperature of + 4 °C (MPW 351, MPW Med. Instruments, Warsaw, Poland). The plasma and the serum were separated from morphotic elements and protected against oxidation (10 µL of 0.5 M BHT/1 mL of serum/plasma). The separated blood components were stored at a temperature of − 80 °C until analysis, but not longer than for six months.

## Methods

### Redox assay

The reagents for the redox assay were supplied by Sigma-Aldrich (Germany) or St. Louis (MO, USA) (until indicated otherwise). Absorbance and fluorescence were measured with the Infinite M200 PRO multi-mode microplate reader (Tecan Group Ltd., Männedorf, Switzerland). Fluorescence was evaluated in black 96-well microplates. All analyses were performed in duplicate. The results were standardized to 1 mg of total protein. Total protein content was determined with a spectrophotometer (Thermo Scientific PIERCE BCA Protein Assay; Rockford, Illinois, USA).

### Nitrosative stress

#### Nitric oxide (NO)

Nitric oxide (NO) concentration was determined indirectly in the Griess reaction by quantifying stable products of NO degradation, i.e. ${\text{NO}}_{2}^{ - }$ and ${\text{NO}}_{3}^{ - }$. Samples of 100 µL each were incubated on 96-well microplates at a temperature of 37 °C for 15 min (500 rpm) with 100 µL of freshly prepared Griess reagent (1% sulfanilamide and 0.1% NEDA · 2 HCl (N-(1-naphthyl)ethylenediamine dihydrochloride in 2.5% methaphosphoric acid). Absorbance was measured in 96-well plates at a wavelength of 490 nm. Nitric oxide concentration was calculated from the calibration curve for NaNO_2_ (0–60 µmol/l)^73,74^.

#### S-nitrosothiols

S-nitrosothiols were quantified in the Griess reaction with Cu^2+^ ions with the use of a spectrophotometer. Samples of 10 µL each were incubated on 96-well microplates at a temperature of 37 °C for 20 min (500 rpm) with 190 µL of the freshly prepared and modified Griess reagent (1% sulfanilamide, 0.1% mM NEDA · 2 HCl (N-(1-naphthyl)ethylenediamine dihydrochloride and 5% CuCl_2_ in phosphate buffered saline solution, pH 7.4). Absorbance was determined at a wavelength of 490 nm with an extinction coefficient e = 11 500 M^−1^ cm^−1^^75^.

#### Nitrotyrosine

Nitrotyrosine levels were determined in a spectrophotometer with the use of a commercial ELISA kit (Immundiagnostik AG; Bensheim, Germany). Samples of 15 µl each were diluted in a 1.5 ml reaction vial, add 885 µl assay buffer (ASYBUF) and mixed (dilution 1:60). Standards, controls and prepared samples are added into the 96—wells of a micro plate coated with polyclonal goat anti- nitrotyrosine antibody. During the first incubation step, nitrated proteins are bound by the immobilised primary antibody. Next a peroxidase-conjugated polyclonal goat anti-human serum proteins antibody is added into each microtiter well and a “sandwich” of primary antibody—nitrated protein—peroxidase-conjugate is formed. Tetramethylbenzidine is used as peroxidase substrate. Finally, an acidic stop solution is added to terminate the reaction. Absorbance was determined at a wavelenght of 450 nm against 620 nm (or 690 nm) as a reference.

### Protein glycoxidation products

#### Tryptophan, kynurenine, N-formylkynurenine, and dityrosine

The concentrations of tryptophan, KN, N-formylkynurenine, and DT were determined in a fluorometric assay. Before the analysis, the samples were diluted in 0.1 M H2SO4 (1:5, v/v) and mixed thoroughly on a vortex for microplates. Characteristic fluorescence was measured in 200 µL of diluted samples in black bottom 96-well plates at a wavelength of 295/340, 365/480, 325/434, and 330/415 nm, respectively. The results were expressed in active-fluorescent units (AFU)/mg of protein^76,77^.

#### Advanced glycation end products

The content of advanced glycation end products (AGEs) in the blood plasma was determined with a spectrofluorometer. The fluorescence emissions of pyrraline, furoyl-furanyl-imidazole (FFI), and carboxymethyl-lysine (CML) were determined at a wavelength of 350/440 nm by measuring the characteristic fluorescence of AGEs^78^. Before the analysis, the samples were diluted in 0.1 M sulfuric acid (1:5, v/v) and mixed thoroughly on a vortex for microplates. Characteristic fluorescence was measured in 200 µL of diluted samples in black bottom 96-well plates^79^.

#### Statistical analysis

Statistical significance was established at *p* < 0.05. The normality of the distribution was evaluated by the Shapiro–Wilk test, and the homogeneity of variance was assessed by Levene’s test. Differences between two groups with multiple variables were compared in a multivariate permutation test. In the absence of normal distribution, differences between two independent groups were compared in the Mann–Whitney U test. The results were presented as median values (minimum–maximum). The diagnostic utility of redox biomarkers was assessed in the Receiver Operating Characteristic (ROC) analysis. The area under the curve (AUC) and the optimal cut-off values were determined for each parameter to ensure high sensitivity and specificity.

The number of participants was based on our previous experiment which involved 15 patients (online ClinCalc software). The concentrations of NO and AGEs were used as variables to calculate sample size. The level of significance was set at 0.05, and the power of the study was 0.9. Group size was determined with the ClinCalc sample size calculator. The minimum number of patients was 48. All assays were performed in duplicate serum samples.

Statistical analyses were performed using GraphPad Prism 9.0 (GraphPad Software, La Jolla, USA) and Past 4.13 (Øyvind Hammer).

### Institutional review board statement

The study was conducted in accordance with the Declaration of Helsinki, and approved by the Bioethics Committee of the Medical University of Białystok (decision No. APK.002.26.2021 of 28 January 2021). All participants gave their written consent to participate in the study for studies involving humans. All data are available from the corresponding author.

### Informed consent

All participants gave their written informed consent to participate in the research.

## Data Availability

The datasets used and/or analysed during the current study available from the corresponding author on reasonable request.
